# Arthroscopic treatment of iliopsoas cyst using outside-in technique—surgical technique and clinical outcomes

**DOI:** 10.1093/jhps/hnaf028

**Published:** 2025-07-12

**Authors:** Zhi Li, Jonathan Patrick Ng, Guanda Lu, Ahamed Fazloon Fathima Farha, Atiya Prajna Hooblal, Timoné Wagner, Fusheng Wang, Weiguo Zhang, Zhiyuan Liang, Kang Tian

**Affiliations:** Department of Joint and Sports Medicine, The First Affiliated Hospital of Dalian Medical University, Lianhe Road 193, Dalian, Liaoning 116011, China; Prince of Wales Hospital, 30-32 Ngan Shing Street, Sha Tin, New Territories 999077, Hong Kong; Department of Joint and Sports Medicine, The First Affiliated Hospital of Dalian Medical University, Lianhe Road 193, Dalian, Liaoning 116011, China; Department of Joint and Sports Medicine, The First Affiliated Hospital of Dalian Medical University, Lianhe Road 193, Dalian, Liaoning 116011, China; Department of Joint and Sports Medicine, The First Affiliated Hospital of Dalian Medical University, Lianhe Road 193, Dalian, Liaoning 116011, China; Department of Joint and Sports Medicine, The First Affiliated Hospital of Dalian Medical University, Lianhe Road 193, Dalian, Liaoning 116011, China; Department of Joint and Sports Medicine, The First Affiliated Hospital of Dalian Medical University, Lianhe Road 193, Dalian, Liaoning 116011, China; Department of Joint and Sports Medicine, The First Affiliated Hospital of Dalian Medical University, Lianhe Road 193, Dalian, Liaoning 116011, China; Department of Joint Surgery and Sports Medicine, Jining First People’s Hospital, Jiankang Road 6, Jining, Shandong 272011, China; Department of Joint and Sports Medicine, The First Affiliated Hospital of Dalian Medical University, Lianhe Road 193, Dalian, Liaoning 116011, China; Prince of Wales Hospital, 30-32 Ngan Shing Street, Sha Tin, New Territories 999077, Hong Kong

## Abstract

Due to the low prevalence of iliopsoas cysts, as well as the technical challenges involved in locating cysts from outside the joint capsule during arthroscopic surgery, there is a lack of comprehensive arthroscopy technique and clinical literature on this topic. We conducted a retrospective analysis and follow-up of five patients with iliopsoas cysts treated arthroscopically, over the past 3 years. Patient ages ranged from 39 to 73 years, with a mean age of 54 years. Our surgical approach involves the utilization of the outside-in technique to access and excise the cysts, along with potential concurrent procedures such as repair or debridement of the acetabular labrum. Each patient had a follow-up duration exceeding 23 months. The outcomes revealed significant pain relief and hip joint function improvement in all patients postoperatively. The Visual Analogue Scale scores reduced from a mean of 5.75 ± 0.43 preoperatively to 1.00 ± 0.70 postoperatively (*P* < .01), and the Modified Harris Hip Score increased from 50.75 ± 4.43 to 86.00 ± 4.47 (*P* < .01). For individuals presenting with symptoms of groin pain in the presence of a radiologically confirmed iliopsoas cyst, hip arthroscopy using the outside-in technique represents a minimally invasive and reliable approach for cyst removal.

## Introduction

Iliopsoas cysts are relatively rare clinically. The exact cause of iliopsoas cysts is still unknown, which may be isolated, or associated with hip pathology, as the iliopsoas bursa often communicates with the hip joint [1]. Iliopsoas cysts present as pain, limping, and tenderness in the groin area, and in some patients, a palpable mass in the groin may be evident, which can also exert compressive symptoms on nerves and blood vessels [2, 3]. However, there is scant clinical literature regarding the treatment of iliopsoas cysts. Arthroscopic intervention for iliopsoas cysts has distinct advantages, offering a less invasive alternative to open surgical procedures. Due to the rarity of this disease along with technical challenges inherent in locating cysts from outside the joint capsule during arthroscopic surgery, there is a lack of surgical technique descriptions and reports of clinical cases. In this article, we will provide a detailed account of our surgical technique and present the clinical follow-up data from our patient series, shedding light on the potential benefits and efficacy of this approach in the management of iliopsoas cysts.

## Materials and Methods

**Figure 1:**
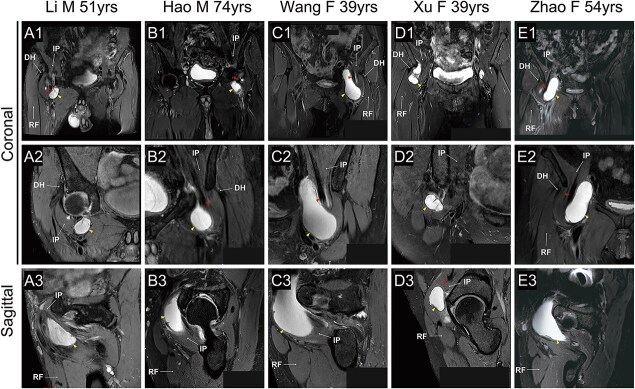
MRI for iliopsoas cysts. Iliopsoas tendon (red arrowhead); iliopsoas cysts (yellow arrowhead); IP, iliopsoas; DH, the direct head of rectus femoris; RF, rectus femoris; M, male; F, female.

Our hospital is a public hospital serving a region of roughly 7.45 million inhabitants. This was a retrospective study looking at consecutive cases in our single institution. All patients fulfilling the criteria of symptomatic iliopsoas cysts, between 2021 and 2024, were included in the study. A total of five patients with clinical symptoms and clear imaging findings were identified; there were no patients excluded from analysis. None of them had a history of trauma or previous hip surgery. They all presented with a chief complaint of local skin bulging in the groin with tenderness and groin pain. Other clinical symptoms included limited hip flexion and internal rotation, and in one case, numbness and decreased muscle strength in the anterior thigh. On exam, 60% (3/5) of the hips were painful with the anterior impingement test. The connecting holes and concurrent labral tears could be seen in all patients by preoperative magnetic resonance imaging (MRI) (Fig. 1). The mean dimensions of the cyst were 65 mm in the coronal view and 30 × 39 mm in the axial view. All procedural consents and details have been approved by the Medical Ethics Committee of the First Affiliated Hospital of Dalian Medical University (PJ-KS-KY-2023-574). Written informed consent has been obtained from patients for publication of this report and any accompanying images.

### Surgical technique

**Figure 2:**
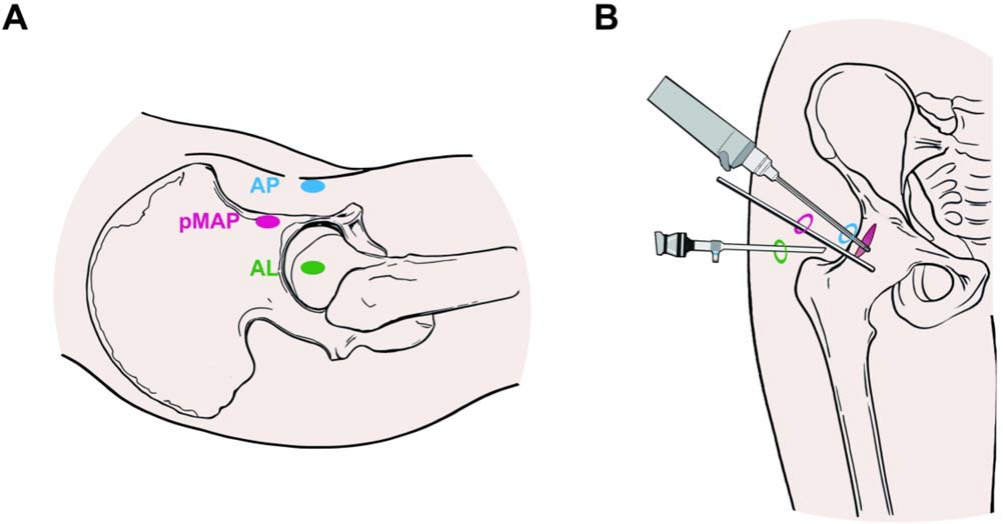
Portal placements and joint capsule incision on a right hip. AL, anteriorlateral portal; AP, anterior portal; pMAP, proximal midanterior portal.

**Figure 3:**
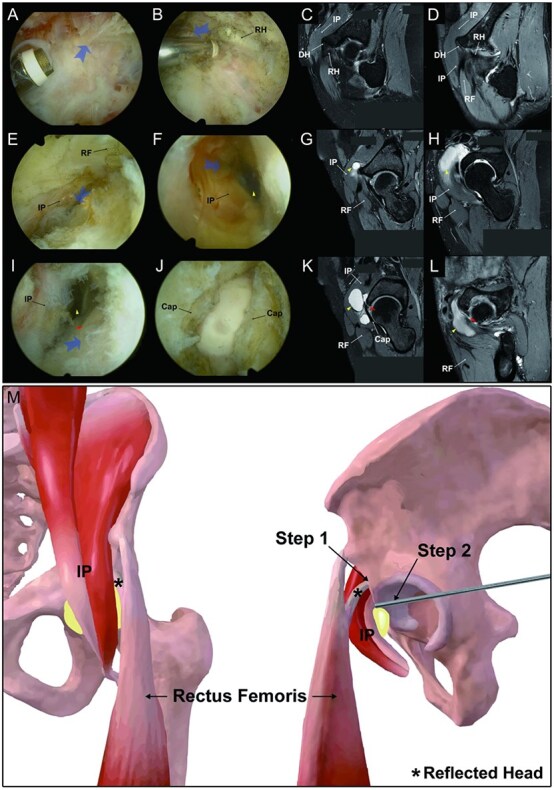
A comprehensive visualization of surgical technique based on arthroscopic view, MRI anatomical annotation, and schematic diagram of surgical approach. (A) Removing the pericapsular fatty tissue (The arrow indicates the areas of exposure; View of hip through a 30° arthroscope in an anterolateral portal and instruments were inserted through an anterior portal), (B-D) then locating the rectus femoris and its reflected head. (E) Lifting the iliopsoas muscle upwards and (F–H) finding the iliopsoas cysts (yellow arrowhead). (I) Locating the connecting holes (red arrow) at the anterior joint capsule (black arrows) and (J–L) performing an outside-in technique. Step 1—locating the reflected head of the rectus femoris (star sign) and step 2—using the exchange rod for lifting the iliopsoas tendon (M). IP, iliopsoas; DH, the direct head of rectus femoris; RH, the reflected head of the rectus femoris; RF, rectus femoris; cap, joint capsule.

The patient is placed in a supine position on a traction table under general anaesthesia. Portal placements are shown in Fig. 2. Using a spinal needle and subsequent trocar, the standard anterolateral portal is placed 1 cm proximal and anterior to the greater trochanter of the femur. The decision to perform intraoperative fluoroscopy for portal placement is based on the surgeon’s proficiency [4, 5]. Once the spinal needle contacts with the hip capsule, clear tactile feedback appears, as signified by an increase in resistance. This ensures that the placement of the portal is extra-articular. The arthroscope is inserted into this extra-articular space for visualization. The standard anterior portal is then crafted in an outside-in fashion under direct arthroscopic visualization. The whole surgical process is shown in Fig. 3. Pericapsular fatty tissue in the anterior aspect of the hip joint capsule is identified and then removed *via* shaver and radiofrequency to create an operative space. Proximally, the reflected head of the rectus femoris is located, and by following it medially, the iliopsoas muscle can be found. An accessory working portal—proximal midanterior portal (pMAP)—is established. The pMAP locates 4 cm proximal to the standard midanterior portal. And a switching stick is employed to lift the iliopsoas muscle upwards, revealing the cystic lesion located on the inner side of the iliopsoas muscle. Typically, a significant amount of yellow viscous fluid is present. The arthroscopic operative space is enlarged to access the cyst and perform excision under arthroscopy. In all cases, the location of communication between the iliofemoral bursa and the joint lies in the damaged anterior joint capsule, which is usually easy to identify during the excision process.

Taking the attachment of reflected head of rectus femoris as an anatomical mark, a longitudinal incision is made using outside-in technique between the gluteus minimus and the iliocapsularis muscles to enter the central compartment of the joint and to evaluate the labral damage. In our cases, labral damage was clearly observed in all patients during surgery. Labral tears were typically located in the 12 o’clock to 2 o’clock region (using the clock face method, where 6 o’clock denoted the transverse ligament and 3 o’clock denoted anterior). Suture repair or debridement of the labrum is carried out according to the labral quality and the status of labral tear. For patients presenting with concomitant osseous structural deformities, acetabular and femoral osteoplasty is performed as indicated during surgery. Closure of the joint capsule is done after all other procedures are completed. Typically, the longitudinal capsular incision measures approximately 3 cm. Generally, we make two stitch-capsular closures for longitudinal incision for patients without high-risk factors of postoperative anterior instability. All patients start a passive range of motion (ROM) rehab 24 h postoperatively, initiate non-weight-bearing active ROM rehab after 48 h, partial weight-bearing at 4 weeks, and full weight-bearing after 6 weeks postoperatively.

## Results

All patients were followed up for at least 23 months after surgery and showed significant improvement in all symptoms without any complications. The clinical data and latest ROM of the patients are shown in Table 1. The Visual Analogue Scale scores reduced from a mean of 5.75 ± 0.43 preoperatively to 1.00 ± 0.70 postoperatively (*P* < .01), and the Modified Harris Hip Score increased from 50.75 ± 4.43 to 86.00 ± 4.47 (*P* < .01) (paired *t*-tests in SPSS 16.0 software). All cysts resolved completely, as confirmed by ultrasound or MRI at the last follow-up, with no evidence of recurrence.

**Table 1:** Clinical data.

	Age (y)	Gender^a^	Additional procedures	Follow-up (mo)	Range of motion (deg) at final follow-up					
					Flexion	Extension	Abduction	Adduction	External rotation	Internal rotation
Li	51	M	Labral repair	23	120	5	40	25	45	30
Hao	74	M	Debridement/Osteoplasty	26	120	0	35	20	45	25
Wang	39	F	Labral repair/Osteoplasty	24	125	10	45	30	50	35
Xu	39	F	Labral repair/Osteoplasty	30	125	10	45	30	50	35
Zhao	54	F	Labral repair	24	120	5	40	30	50	30

a M, male; F, female.

## Discussion

We present our experience in utilizing hip arthroscopy with the outside-in technique for the treatment of iliopsoas cysts and report the clinical outcomes of five patients. The treatment of iliopsoas cysts can involve nonsteroidal anti-inflammatory drugs, ultrasound-guided puncture, and surgical treatment. McGill *et al.* presented the standard technique for ultrasound-guided puncture [6]. However, compared to arthroscopic surgery, ultrasound-guided puncture, although less invasive, can only temporarily relieve local pressure because it does not remove the cyst wall, resulting in a higher recurrence rate. Di Benedetto *et al.* and Di Sante *et al.* independently reported cases where the cyst recurred quickly after puncture and aspiration and was successfully treated with surgery [7, 8]. The anteromedial aspect of the iliopsoas tendon is where the femoral vessels and nerve are located. Our surgical approach was to proceed from the outside of the hip joint, to the iliopsoas muscle, and then to the joint cavity. This arthroscopic excision of the cyst avoids direct interference with blood vessels and nerves, making it safer than open surgery. We describe the meaningful landmarks from the arthroscopic perspective for surgeon to identifiable arthroscopically and enable safer and more efficient surgery in this uncommonly accessed anatomic region. The described technique is quick and technically easy to learn. A fixed viewing portal and two working portals were established. The portals were at a certain distance from each other to allow proper triangulation. During the surgery, the reflected head of the rectus femoris was located, and the joint capsule was cleaned medially along the reflected head. The exchange rod through the proximal midanterior portal approach could lift the iliopsoas tendon, allowing easy identification of the iliopsoas bursal cyst and insertion of the shaver and radiofrequency probe into the cyst to clean the cyst wall, which is crucial for preventing postoperative recurrence. The rupture site of the joint capsule could be seen behind the iliopsoas tendon, where the joint capsule was incised, and sutured after addressing intra-articular pathology. Potential complications include stiffness and pain of the anterior hip, although we have never encountered these. The advantages and disadvantages are summarized in Table 2.

**Table 2:** Technical pearls and pitfalls.

**Pearls**		**Pitfalls**	
**Description**	**Advantages**	**Description**	**Avoidance strategy**
Outside-in approach: Reflected head of rectus femoris → iliopsoas → cyst → joint cavity.	This improves cyst exposure, avoiding additional lesions in anterior soft tissue.	Latrogenic injury of labrum during cutting	The cut is made from distal to proximal, following the direction of the fibres; Progressively detaching to optimize labral exposure and visualization; Aided positioning by connecting holes

## Conclusion

This technique is characterized by minimal trauma, safe and reproducible, and can simultaneously address both intra-articular and extra-articular diseases. Therefore, for large iliopsoas cysts that produce symptoms, especially those with associated intra-articular pathology, hip arthroscopy using the outside-in technique will be a reliable approach.

## Data Availability

The data underlying this article will be shared upon reasonable request to the corresponding author.
